# The Easy Mark

**Published:** 1902-08

**Authors:** 


### THE EASY MARK.

The following is from *The Chicago Clinic and Pure Water Journal:*

"The latest hospital fad is a proposal to supply each public school with one or more hospital cots and emergency remedies in case of sudden sickness on the part of teachers or scholars. We suggest that the room be equipped with blue glass and a Roentgen ray apparatus and that the leading physicians of the place be allowed to furnish continuous services without pay."

To this we may add, that if the *Clinic's* suggestion be carried out, in all probability, as soon as the opportunity is given them, the "leading physicians" will make a bargain counter rush to proffer their servises, and for the sake of improving their chances of being selected most of them will no doubt be glad to supply the necesary cots and medicine at their own expense. R.

